# Coculture of bovine cartilage with synovium and fibrous joint capsule increases aggrecanase and matrix metalloproteinase activity

**DOI:** 10.1186/s13075-017-1318-9

**Published:** 2017-07-05

**Authors:** Per Swärd, Yang Wang, Maria Hansson, L. Stefan Lohmander, Alan J. Grodzinsky, André Struglics

**Affiliations:** 1 Orthopedics, Department of Clinical Sciences Lund, Lund University, BMC C12, SE-221 84 Lund, Sweden; 2 Department of Biological Engineering, Massachusetts Institute of Technology, Cambridge, MA USA; 3 Department of Mechanical Engineering, Massachusetts Institute of Technology, Cambridge, MA USA; 4 Department of Electrical Engineering, Massachusetts Institute of Technology, Cambridge, MA USA

**Keywords:** Aggrecan, Cartilage injury, Fibrous joint capsule, Knee injury, Synovium, Metalloproteinases, Aggrecanases

## Abstract

**Background:**

A hallmark of osteoarthritis is increased proteolytic cleavage of aggrecan. Cross talk between cartilage and the synovium + joint capsule (SJC) can drive cartilage degradation by activating proteases in both tissues. We investigated aggrecan proteolysis patterns in cartilage explants using a physiologically relevant explant model of joint injury combining cartilage mechanical compression and coincubation with SJC.

**Methods:**

Bovine cartilage explants were untreated; coincubated with SJC; or subjected to mechanical injury and coincubated with SJC, mechanical injury alone, or mechanical injury and incubated with tumor necrosis factor-α (TNF-α). To compare the patterns of aggrecan proteolysis between 6 h and 16 days, release of sulfated glycosaminoglycans and specific proteolytic aggrecan fragments into medium or remaining in cartilage explants was measured by dimethylmethylene blue and Western blot analysis.

**Results:**

Aggrecanase activity toward aggrecan was observed in all conditions, but it was directed toward the TEGE↓ARGS interglobular domain (IGD) site only when cartilage was coincubated with SJC or TNF-α. Matrix metalloproteinase (MMP) activity at the aggrecan IGD site (IPES↓FFGV) was not detected when cartilage was exposed to TNF-α (up to 6 days), but it was in all other conditions. Compared with when bovine cartilage was left untreated or subjected to mechanical injury alone, additional aggrecan fragment types were released into medium and proteolysis of aggrecan started at an earlier time when SJC was present.

**Conclusions:**

Indicative of different proteolytic pathways for aggrecan degradation, the SJC increases both aggrecanase and MMP activity toward aggrecan, whereas TNF-α inhibits MMP activity against the IGD of aggrecan.

**Electronic supplementary material:**

The online version of this article (doi:10.1186/s13075-017-1318-9) contains supplementary material, which is available to authorized users.

## Background

Articular cartilage is a dynamic tissue in synovial joints that can withstand substantial loads within physiological levels [1, 2]. Aggrecan, the major proteoglycan of cartilage, is substituted with negatively charged sulfated glycosaminoglycans (sGAGs) and forms large aggregates by binding to hyaluronan [3]. The negative charge density induces a high osmotic swelling pressure within cartilage, restrained by the collagen fibril network [3]. These osmotic and electrostatic repulsive interactions provide more than 50% of cartilage’s equilibrium compressive modulus. In addition, the closely spaced sGAG chains resist fluid flow caused by dynamic compression quantified at both the molecular level [4] and the tissue level [5]. The resulting intratissue pressurization caused by dynamic compression greatly increases cartilage’s dynamic modulus at high loading rates. Proteolytic cleavage of aggrecan is an important feature in osteoarthritis (OA) [6–8]. Aggrecanase-1 and aggrecanase-2 (a disintegrin and metalloproteinase with thrombospondin motifs 4 and 5 [ADAMTS4 and ADAMTS5, respectively]) and the matrix metalloproteinases (MMP-1, MMP-3, MMP-8, MMP-9, and MMP-13) are believed to be the most important enzymes responsible for the proteolytic degradation of aggrecan [9].

A severe joint injury leads to an increased risk of developing posttraumatic osteoarthritis (PTOA) [10]. PTOA pathogenesis is multifactorial, and many risk factors associated with developing nontraumatic OA, such as age, obesity, and genetic variation, may also apply to PTOA [10]. With regard to knee OA, the risk is related to the integrity of the menisci; to associated compressive injuries to ligaments, cartilage, and bone; and to joint synovitis [11–14]. In the acute phase of injury, impact forces applied over the knee joint in combination with hemarthrosis lead to activation and recruitment of immune cells to the knee joint, producing an inflammatory and procatabolic joint environment. Several reports have demonstrated rapid increases in pro- and anti-inflammatory cytokines, proteases, and proteolytic activity after a severe knee injury [15–22]. Cells of the synovium and fibrous joint capsule (referred to hereafter as the *synovium + joint capsule* [SJC]), comprising resident inflammatory cells, synovial cells, and other cells such as endothelial cells and blood leukocytes, contribute to joint inflammation by producing proinflammatory mediators and proteases such as aggrecanases and MMPs [23, 24]. Matrix molecule fragments and cell debris released into the joint after trauma lead to activation of various cells in the cartilage and the SJC, further increasing the proinflammatory response [24]. In the long term, imbalance between pro- and anticatabolic activities may lead to progressive cartilage extracellular matrix degradation and development of OA [15, 19, 25–29].

In the present in vitro study, we investigated differences in the preferred enzymatic cleavage of aggrecan in bovine cartilage explants cocultured with or without SJC, cartilage exposed to mechanical trauma, or cartilage exposed to exogenous tumor necrosis factor-α (TNF-α). We hypothesized that in this coculture system, there is cross talk between traumatized cartilage and the SJC through cell- and matrix-derived factors, leading to increased aggrecanase and MMP cleavage of the cartilage aggrecan. To quantify aggrecanase and/or MMP activity toward aggrecan in these different conditions, we used a set of well-characterized antibodies directed at different cleavage sites in the aggrecan molecule.

## Methods

### Bovine cartilage and SJC harvesting

The methods of cartilage explant acquisition for the experiment are described elsewhere [30]. In brief, articular cartilage discs were harvested from the femoropatellar grooves of 1- to 2-week-old calves obtained on the day of slaughter (Research 87, Boylston, MA, USA). Full-thickness cartilage cylinders were cored using a 3-mm dermal punch, and the top 1-mm disc containing an intact superficial zone was harvested with a blade. Discs were preincubated in serum-free medium (low-glucose DMEM [1 g/L]) supplemented with 10 mM HEPES buffer, 0.1 mM nonessential amino acids, 0.4 mM proline, 20 μg/ml ascorbic acid, 100 U/ml penicillin G, 100 μg/ml streptomycin, and 0.25 μg/ml amphotericin B for 2–3 days (5% CO_2_, 37 °C). Full-thickness explant discs inclusive of the fibrous joint capsule and the synovium were harvested [26] from the same animals’ knees using a 5-mm dermal punch and were preincubated separately in serum-free medium for 2–3 days. The tissue consisted of the fibrous joint capsule with a single layer of synovium varying in thickness from 0.5 to 3 mm [31].

### Cartilage injury and treatment conditions

After preequilibration, six cartilage discs per condition were treated according to one of the following conditions: (A) untreated (uninjured cartilage), (B) mechanically injured (injured cartilage), (C) coincubated with SJC (uninjured cartilage + SJC), (D) mechanically injured and coincubated with SJC (injured cartilage + SJC), or (E) mechanically injured and incubated with exogenous TNF-α at 25 ng/ml (injured + TNF-α), and (F) six SJC explants were also cultured alone (SJC alone) [32]. Tissues and medium were removed from culture at different incubation times ranging from 6 h to 16 days (Table 1). Explant and SJC samples were harvested from six joints of three animals. One cartilage explant from one joint of one animal was cultured alone (conditions A, B, and E) or in the same well as an SJC explant from the same joint (conditions C and D). In condition F, separate SJC specimens of were cultured. There were 6 cartilage explants used for each time point, giving us 36 cartilage explants for the 6 time points in each condition (A–D) plus separate specimens for SJC (conditions C and D). In conditions E and F, six explants were cultured for 6 and 16 days, respectively. Medium was changed (300 μl) after 6 h and 2 days of incubation and thereafter every 2 days until the end of incubation (Table 1).

**Table 1:** Culture conditions A–F and harvesting time after incubation start

Conditions	Sample collection, days of incubation	
	Medium	Tissue
Uninjured cartilage (A)	0.25, 1, 2, 4, 6, 8, 10, 12, 14, 16	0.25, 1, 2, 4, 6, 16
Injured cartilage (B)	0.25, 1, 2, 4, 6, 8, 10, 12, 14, 16	0.25, 1, 2, 4, 6, 16
Uninjured cartilage + SJC (C)	0.25, 1, 2, 4, 6, 8, 10, 12, 14, 16	0.25, 1, 2, 4, 6, 16
Injured cartilage + SJC (D)	0.25, 1, 2, 4, 6, 8, 10, 12, 14, 16	0.25, 1, 2, 4, 6, 16
Injured cartilage + TNF-α (E)	0.25, 1, 2, 4, 6	6
SJC alone (F)	0.25, 1, 2, 4, 6, 8, 10, 12, 14, 16	16

*Abbreviations: SJC* Synovium + joint capsule, *TNF-α* Tumor necrosis factor-α

In conditions involving mechanical injury, injurious unconfined compression to a final strain of 50% at a strain rate of 100%/second was applied to the cartilage (conditions B and D) using a custom-designed loading apparatus just prior to the start of culture [30, 33]. Purified bovine aggrecan (A1D1 fraction, *see* below) was incubated (0.22 μg sGAG/μl) at 37 °C in serum-free medium for 1 h or 24 h in the presence or absence of bovine SJC (67 μg wet wt/μl) and TNF-α (10 pg/ml). After incubation, the aggrecan solution and tissue were frozen separately.

### Purification of proteoglycans and aggrecan

Proteoglycans (including full-length aggrecan and proteolytic fragments of aggrecan) were purified by guanidine extraction from all cartilage discs and SJCs. The discs (approximately 10 mg each) were extracted by stirring at 4 °C in a total of 17 μl of extraction buffer (50 mM Na^+^-acetate, 4 M guanidinium HCl, 10 mM ethylenediaminetetraacetic acid [EDTA], 100 mM 6-aminocaproic acid, 10 mM *N*-ethylmaleimide, 5 mM benzamidine HCl, 1 mM phenylmethanesulfonyl fluoride, pH 6) per milligram of tissue. Thereafter, the guanidine-extracted proteoglycan sample was centrifuged (20,000 × *g*, 20 minutes at 4 °C) to collect the supernatant, which was precipitated for 1 h at −20 °C with 5 vol of ice-cold acetone. The proteoglycan pellets were then collected by centrifugation (20,000 × *g*, 20 minutes at 4 °C) and dissolved in deglycosylation buffer (50 mM Tris-acetate, 50 mM Na^+^-acetate, 10 mM EDTA, pH 7.6). In separate tests, aggrecan was purified from freshly harvested, untreated bovine calf femoropatellar groove cartilage samples that were immediately frozen after removal from the same knee joints as the treated cartilage explants. Briefly, proteoglycans were first purified by guanidine extraction (as described above), and aggrecan was then purified by CsCl density centrifugation to collect the A1D1 fraction [34].

### Analysis of sGAG and aggrecan

Culture medium, guanidine-extracted proteoglycan samples from cartilage, and SJCs were analyzed for sGAG content using 1,9-dimethylmethylene blue (DMMB) [35], with the following changes: 20-μl samples or standards (chondroitin sulfate [CS], catalogue number C4384; Sigma-Aldrich, St. Louis, MO, USA) were mixed on a 96-well microtiter plate with 200 μl of DMMB, and absorbance was measured at 520 nm with a plate reader.

For Western blot analysis, samples were deglycosylated as described elsewhere [8], with the exception that incubation was performed with lower keratanase II concentrations but for a longer time with chondroitinase ABC (EC 4.2.2.4, 2 h at 37 °C, 1 mU/mg sGAG; Sigma-Aldrich), keratanase (EC 3.2.1.103, 1 h at 37 °C, 1 mU/mg sGAG; Seikagaku, Tokyo, Japan), and keratanase II (*Bacillus* sp. Ks36, 3 h at 37 °C, 0.01 mU/mg sGAG; Seikagaku). Deglycosylated samples were precipitated with 5 vol of ice-cold acetone, and proteins were collected by centrifugation (20,000 × *g*, 20 minutes at 4 °C) and dissolved in 40–50 μl of 2× concentrated sample buffer (SB) (NuPAGE Novex; Life Technologies, Carlsbad, CA, USA) in the following conditions: 4 μl of medium/1 μl of SB, 10–150 μg wet wt cartilage/1 μl of SB, and 80–200 μg wet wt SJC/1 μl of SB.

For quantitative Western blotting of aggrecan fragments, purified calf cartilage aggrecan A1D1 fraction (8 standard points per gel; 0.02–0.48 μg of sGAG loaded per well) was used as the standard for aggrecan G3 fragments [36]. Calf cartilage aggrecan A1D1 fraction (500 μg) was digested for 24 h by ADAMTS4 (0.5 μg) [37] (6 standard points per gel; 0.01–0.36 μg of sGAG loaded per well) and was used as the standard for ARGS-aggrecan fragments. Calf cartilage aggrecan A1D1 fraction (100 μg) was digested for 24 h by MMP-3 (1 μg) [38] (7 standard points per gel [0.002–0.018 μg of sGAG loaded per well]) and was used as the standard for FFGV-aggrecan fragments. Because KEEE standards were not available, calf cartilage aggrecan A1D1 fraction (80 μg) was digested 10 minutes with ADAMTS4 (0.08 μg) for use as a control sample (1 μg of sGAG loaded per well three times) for quantification of KEEE fragments, expressing the data as arbitrary units of the control sample. All standards were deglycosylated. FFGV, ARGS, KEEE, and G3 aggrecan fragments were quantified by Western blot analysis (using the LAS-1000 imaging system and Image Gauge version 3.2 software [FUJIFILM Medical Systems, Stamford, CT, USA], with settings of regions of interest done by one experienced operator [MH]) in medium samples (i.e., six samples per time point and condition). On the basis of the film images of these experiments, an experienced operator (AS) determined the presence and absence of bands and the time point when a band was first observed.

**Fig. 1:**
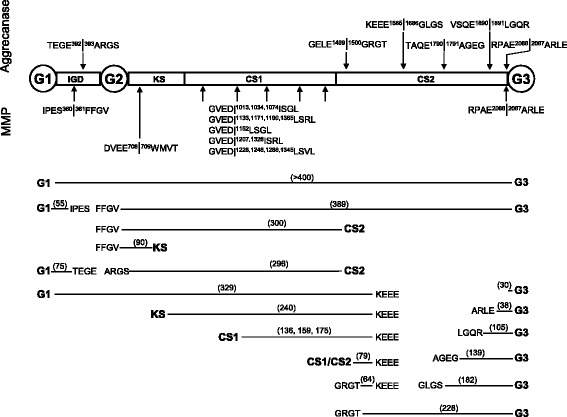
Matrix metalloproteinase (MMP) and aggrecanase cleavage sites in bovine aggrecan. Aggrecanase and MMP cleavage sites of bovine aggrecan were identified by amino acid sequence comparison with human aggrecan sequence by Western blot analysis of bovine aggrecan (*see* Additional file 1: Figure S1) and as previously described [38, 47, 54–56]. Bovine aggrecan fragments, with molecular mass in kilodaltons in brackets, detected in explant cultures are shown. Using GVEDI(L)SG(R/V)L as a consensus sequence for MMP digestion resulted in 14 potential cleavage sites for MMP in the bovine CS1 region. The amino acid numberings are based on the full-length bovine aggrecan amino acid sequence starting with the N-terminal ^1^MTTL and finishing with the C-terminal STAH^2364^ (National Center for Biotechnology Information accession number P13608). *IGD* Interglobular domain, *KS* Keratan sulfate region, CS Chondroitin sulfate region, G1–G3, Globular domains 1–3

Immunoreactions were done [34] using anti-ARGS (neoepitope monoclonal OA-1 antibody, 5.3–14 μg/ml; GlaxoSmithKline), anti-AGEG (neoepitope sera, 1:500; GlaxoSmithKline), anti-FFGV (neoepitope monoclonal AF-28 antibody, 0.5 μg/ml; EMD Millipore, Billerica, MA, USA), anti-G3 (polyclonal antibody, 5 μg/ml, PA1-1745; Pierce Biotechnology, Rockford, IL, USA), anti-KEEE (neoepitope sera, 1:1000), anti-LGQR (neoepitope sera, 1:500), anti-ARLE (neoepitope sera, 1:2000), anti-G1 (sera, 1:5000) against G1 sequence ATEGQVRVNSIYQDKVSL, together with secondary peroxidase-conjugated antibodies of horse antimouse immunoglobulin G (IgG) (1:10,000–1:25,000, catalogue number 7076; Cell Signaling Technology, Danvers, MA, USA) and goat antirabbit IgG (20 ng/ml, catalogue number 074-1516; Kirkegaard & Perry Laboratories, Gaithersburg, MD, USA). The immunobands were visualized using ECL 2 Western Blotting substrate (Pierce Biotechnology) together with a luminescence image analyzer (LAS-1000) and film (Amersham Hyperfilm ECL; GE Healthcare Life Sciences, Little Chalfont, UK). Western blotting was done to verify immunoband blocking of antibody reactions [36]. All the bovine aggrecan fragments detected by Western blot analysis in the medium and cartilage plugs in this study are described in Additional file 1 and are illustrated (Fig. 1 and Additional file 1: Figure S1).

### Statistics

The ratios of FFGV, ARGS, G1-KEEE, and G1–G3 fragments in medium to the total amount of aggrecan (sGAG left in the cartilage explant plus sGAG released into the medium during the 16 days of culture) in the explant system were calculated. The total amount of sGAG released into the medium was calculated as the cumulative amount of sGAG detected in the medium changed every 2 days over the 16 days of incubation. Because of the low number of animals (*n* = 3 cows), a normal distribution was assumed but not tested. All tests were performed using the paired-samples Student’s *t* test of statistical significance (IBM SPSS Statistics version 20 software; IBM, Armonk, NY, USA). A *P* value <0.05 was considered statistically significant. The number of animals used in the statistical analysis was 3, where *n* = 1 was defined as the mean amount of aggrecan fragment from two cartilage explants (one from each separate knee joint) of the same cow. This approach accounts for intra- and interanimal variability. When the quantified amount was below the limit of detection, it was set to half the lowest standard point before statistical testing.

## Results

### Aggrecan left in cartilage or released into medium

**Fig. 2:**
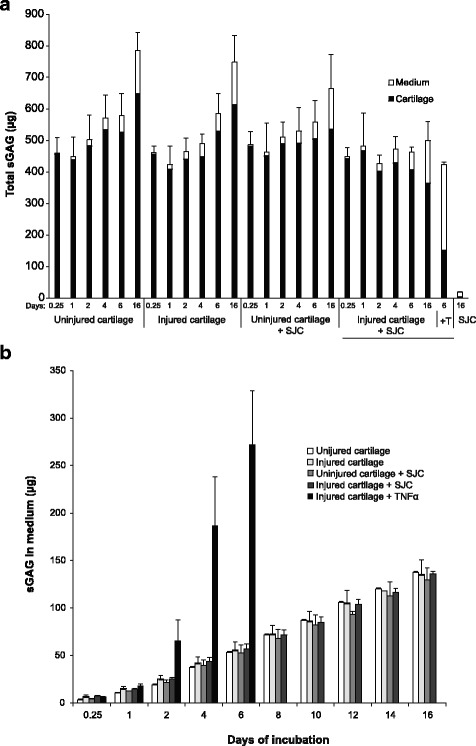
Amount of sulfated glycosaminoglycan (sGAG) in cartilage and medium during explant culturing. **a** Total amount of sGAG in the explant system; cartilage and medium combined over 16 days of incubation. **b** The accumulated amount of sGAG released into medium during the 16 days of culture. The cartilage and synovium + joint capsule (SJC) explants (*n* = 3) were cultured for from 6 h to 16 days. Number of days of culture and sGAG amounts (in mean and SD) are presented. *T, TNF-α* Tumor necrosis factor-α

We used sGAG as a measure of total aggrecan content. The amount of sGAG extracted from the SJC or released into medium from SJC explants was very low: approximately 200-fold less than cartilage explants (Fig. 2a). Hence, the contribution of aggrecan from the SJC to the explant system was considered negligible in the present study.

The total amount of sGAG (sum of what was left in cartilage and released into the medium) increased from 6 h to day 16 in conditions A (uninjured cartilage; 1.7-fold), B (injured cartilage; 1.6-fold), and C (uninjured cartilage + SJC; 1.4-fold), but not in condition D (injured cartilage + SJC; 0.95-fold) (Fig. 2a), suggesting de novo synthesis of aggrecan in conditions A–C during the culture period. However, full-length G1–G3 aggrecan present in the cartilage, quantified by Western blot analysis, decreased by between 57% and 66% from 6 h to day 16 in all conditions (data not shown). This suggests that a substantial fraction of full-length aggrecan present at the start of the experiment was either released from the cartilage or C-terminally truncated, thus missing the G3 domain.

The accumulated release of sGAG into medium over the 16 days of incubation was similar in conditions A–D (with or without injury, with or without SJC). After 6 days of culture, the release of sGAG into medium was approximately 50 μg in conditions A–D, whereas the corresponding amount for condition E (injured cartilage + TNF-α) was substantially higher at about 270 μg (*P* ≤ 0.022 for all) (Fig. 2b). In summary, culture of cartilage explants over 16 days was associated with de novo aggrecan production, except for conditions where cartilage was mechanically injured and cultured in the presence of SJC.

### Enzymatically cleaved aggrecan fragments detected in medium

Although total amounts of bovine aggrecan released from the cartilage into the medium (approximated as total sGAG) did not differ between the culture conditions A–D (Fig. 2b), analysis of the time-dependent release of bovine aggrecan fragments revealed striking differences between these conditions (Table 2 and Additional file 2: Figure S2). Aggrecan fragments with different proteolytic cleavage sites were generated and released into the medium when the cartilage was coincubated with SJC (i.e., 18 fragments in condition C and 19 fragments in condition D compared with without SJC [A and B] with 12 fragments in each condition) (Table 2). For example, aggrecanase-generated ARGS and ARLE-G3 fragments were observed only in medium from culture conditions in which SJC was present or when exogenous TNF-α was added (E). MMP-generated FFGV fragments were observed in all culture conditions, except when exogenous TNF-α was added (E) (Table 2 and Additional file 2: Figure S2). Conditions in which injury was applied to cartilage (B and D) did not generate different types of aggrecan fragments compared with conditions without applied injury (A vs. B and C vs. D).

**Table 2:**
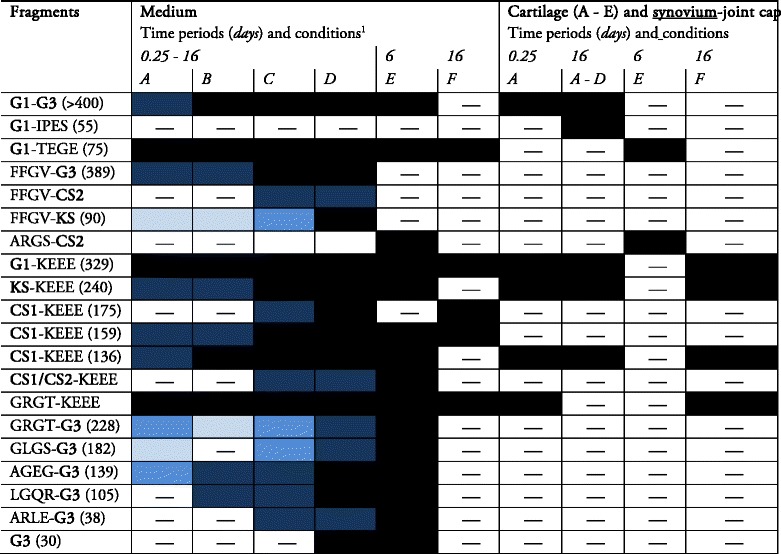
Aggrecan fragments detected by Western blot analysis of bovine explant cultures

1) A = uninjured cartilage; B = injured cartilage; C = uninjured cartilage + synovium-joint capsule; D = injured cartilage + synovium-joint capsule; E = injured cartilage + TNF-α; F = synovium-joint capsule

White to black graded boxes = time periods in days when aggrecan fragments were first observed in medium:

Yellow boxes = detected aggrecan fragments without information when fragments were first observed:

Boxes marked with a straight line ‘—’ = aggrecan fragments not detected

Together, these results suggest that coincubation with SJC generates an increased number of different types of aggrecan fragments compared with conditions without SJCs and that cartilage injury does not increase the number of different aggrecan fragments.

### Initiation and preferred order of enzymatic aggrecan cleavage

Compared with culture conditions without SJC (A and B), cartilage cultured in the presence of SJC (C and D) showed a more rapid aggrecan degradation, observed as an earlier release of several types of aggrecan fragments into the medium (Table 2). No clear differences in the initiation or the preferred order of cleavage of aggrecan were detected between untreated and mechanically injured cartilage (A and B). For all explant conditions, except when injured cartilage was cultured in the presence of TNF-α (E), the earliest aggrecan cleavage was found at the aggrecanase sites GELE↓GRGT and KEEE↓GLGS in the CS2 region, based on analysis of the GRGT-KEEE and G1-KEEE fragments, and at the MMP site IPES↓FFGV in the interglobular domain (IGD), based on analysis of the FFGV-G3 fragment. Cuts at these sites were observed 6 h to 1 day after the start of the incubation (Table 2). Early aggrecan digestion (6 h to 2 days) was also observed for the MMP cuts in the CS1 region (based on analysis of the CS1-KEEE fragments). The cleavage of aggrecan at the aggrecanase site TEGE↓ARGS in the IGD was detected much later, after 6–8 days of incubation (C and D). Together, these results suggest that in these explant systems, aggrecan degradation starts earlier in time in the presence of SJC, and the most preferred cleavage sites are the aggrecanase sites in the CS2 regions and the MMP site in the IGD, whereas the least preferred cleavage site is the aggrecanase site in the IGD.

### Quantitative analyses of aggrecanase- and MMP-generated aggrecan fragments in medium

**Fig. 3:**
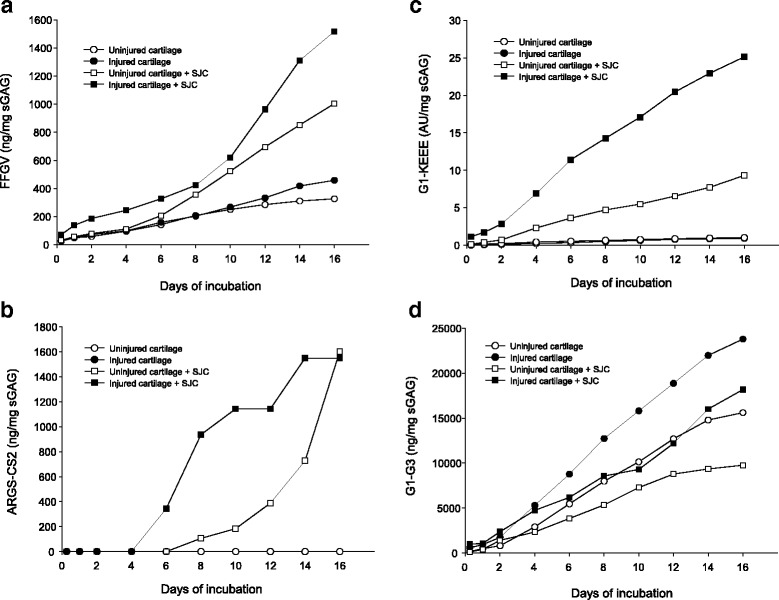
Time-dependent release of aggrecan fragments. The mean accumulated amount of total FFGV (**a**), ARGS-CS2 (**b**), G1-KEEE (**c**), and G1–G3 (**d**) quantified by Western blot analysis in medium over 16 days of incubation. *Open circles*, uninjured cartilage; *filled circles*, mechanically injured cartilage; *open squares*, uninjured cartilage cocultured with synovium + joint capsule (SJC); *filled squares*, mechanically injured cartilage coincubated with SJC. For clarity, no error bars are shown; instead, the individual values are shown in Additional file 3: Table S1 *sGAG* Sulfated glycosaminoglycan

After 16 days of culture, the mean accumulated release of FFGV into medium was higher from injured cartilage (B, 1.4-fold) and from injured cartilage cocultured with SJC (D, 4.6-fold) than from uninjured cartilage (A) (Table 3, Fig. 3). The mean accumulated release of ARGS was 4.8-fold higher from injured cartilage cocultured with SJC than the release from uninjured cartilage (D vs. A) and 4.6-fold higher than the release from injured cartilage (D vs. B) (Table 3, Fig. 3b). Owing to large variations in ARGS concentrations between the cartilage plugs (Additional file 3: Table S1), no statistically significant differences were observed between uninjured cartilage cocultured with SJC (C) and conditions A and B (Table 3), even though visual differences were observed on the Western blots (Fig. 3b, Additional file 2: Figure S2). The mean accumulated release of G1-KEEE was higher in conditions C (9- and 10-fold) and D (28- and 25-fold) than in condition A and condition B (Table 3, Fig. 3c). There were no statistically significant differences in the mean accumulated release of G1–G3 between any of the conditions (Table 3, Fig. 3d).

**Table 3:** Mean (SD) cumulative levels of aggrecan fragments detected in medium after 16 days of culture

FFGV (ng/μg sGAG)	Condition A			Condition B			Condition C			Condition D
	0.326 (0.016)			0.447 (0.036)			1.002 (0.359)			1.506 (0.359)
		*P* value	Norm							
	B vs A	**0.021**	1.37		*P* value	Norm				
	C vs A	0.428	3.07	C vs B	0.509	2.24		*P* value	Norm	
	D vs A	**0.049**	4.62	D vs B	**0.067**	3.37	D vs C	0.744	1.50	
ARGS-CS2 (ng/μg sGAG)	Condition A			Condition B			Condition C			Condition D
	0.48 (0.036)			0.51 (0.056)			2.10 (1.608)			2.32 (1.134)
		*P* value	Norm							
	B vs A	0.193	1.06		*P* value	Norm				
	C vs A	0.254	4.38	C vs B	0.255	4.12		*P* value	Norm	
	D vs A	**0.032**	4.83	D vs B	**0.033**	4.55	D vs C	0.850	1.10	
G1-KEEE (AU/mg sGAG)	Condition A			Condition B			Condition C			Condition D
	0.909 (0.604)			0.986 (1.028)			9.297 (0.781)			25.118 (13.842)
		*P* value	Norm							
	B vs A	0.905	1.08		*P* value	Norm				
	C vs A	**0.011**	10.23	C vs B	**0.016**	9.43		*P* value	Norm	
	D vs A	0.119	27.63	D vs B	0.106	25.47	D vs C	0.298	2.70	
G1–G3 (ng/μg sGAG)	Condition A			Condition B			Condition C			Condition D
	15.3 (2.75)			23.6 (18.27)			9.8 (1.51)			18.6 (14.59)
		*P* value	Norm							
	B vs A	0.460	1.54		*P* value	Norm				
	C vs A	0.159	0.64	C vs B	0.351	0.42		*P* value	Norm	
	D vs A	0.935	1.22	D vs B	0.199	0.79	D vs C	0.603	1.90	

*Abbreviations*: *FFGV* Total levels of FFGV fragments, *Norm* Normalizing mean values. *Conditions: A* Uninjured cartilage, *B* Injured cartilage, *C* Uninjured cartilage + synovium + joint capsule, *D* Injured cartilage + synovium + joint capsule

Statistical (using paired samples *t* test) significance (*P* < 0.05) or trends (*P* < 0.1) are shown in bold

These results together suggest that increased levels of aggrecanase-generated ARGS and KEEE fragments, as well as MMP-generated FFGV fragments, are released into medium when cartilage is coincubated with SJC compared with when SJC is absent. An increased release of MMP-generated FFGV fragments into medium was also observed when cartilage was mechanically injured compared with when cartilage was left untreated.

### The SJC produces active aggrecanases and MMPs

**Fig. 4:**
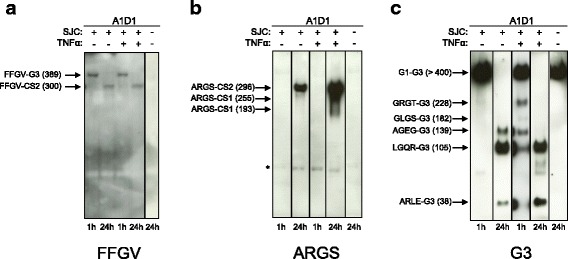
Incubation of aggrecan with synovium + joint capsule (SJC). Bovine aggrecan A1D1 fraction (purified from cartilage) was incubated for 1 h or 24 h with or without bovine SJC in the presence or absence of externally added tumor necrosis factor-α (TNF-α) (*see* Methods section of main text). After incubation, the SJC was removed and aggrecan samples were deglycosylated and run on 3–8% Tris-acetate SDS gels, and immunobands were visualized by Western blotting using FFGV (**a**), ARGS (**b**), and G3 (**c**) aggrecan antibodies. Bovine aggrecan fragments discussed in the text are shown on the *left side* in each panel with the molecular mass indicated in kilodaltons. Representative Western blot images from full-size blotted gels are shown. Sulfated glycosaminoglycan (sGAG) was loaded at 2–3 μg/lane. *Bands considered to be false-positive immunobands because they could not be blocked by their corresponding immunopeptides

Incubation of purified bovine aggrecan with bovine SJC for 1 h induced aggrecan cleavage at the MMP site IPES↓FFGV in the IGD, seen as an FFGV-G3 fragment (Fig. 4a), and cleavage at the aggrecanase site GELE↓GRGT in the CS2 region, seen as a weak GRGT-G3 fragment (Fig. 4c). Incubation for 24 h further processed the FFGV-G3 to a FFGV-CS2 fragment (Fig. 4a), induced cleavage at the aggrecanase site TEGE↓ARGS in the IGD, seen as ARGS-CS1/CS2 fragments (Fig. 4b), and further induced aggrecanase cleavage in the CS2 region, generating AGEG-G3, LGQR-G3, and ARLE-G3 fragments (Fig. 4c). Addition of TNF-α to these incubations increased the aggrecanase activity, seen either as faster generation of fragments (AGEG-G3 and LGQR-G3) (Fig. 4c) or as more intense immunobands (ARGS-CS1/CS2 and GRGT-G3) (Fig. 4b and c). This increase in protease activity was not observed for MMP activity (Fig. 4a). These results suggest that the SJC produces both active aggrecanases and MMPs and that addition of TNF-α increases the induction of aggrecanases.

## Discussion

Proteolysis of human aggrecan showing the fragmentology of aggrecan has been described previously [8, 9, 34, 38], but details such as those in this study, showing aggrecan fragments present in cartilage and released into medium, have not been described to date, to our knowledge. Our findings suggest that coincubation of cartilage with SJC leads to increased proteolytic activity of both MMPs and aggrecanases against aggrecan. These quantitative findings were supported by the qualitative analysis of aggrecan fragments in medium, showing that conditions where the SJC was present were associated with additional enzymatically cleaved aggrecan fragment types released into medium and that proteolytic cleavage of aggrecan started earlier in time in these conditions. Mechanical injury of cartilage alone was associated with a small increase in the amount of FFGV fragments released into the medium, suggesting increased MMP activity toward aggrecan compared with the uninjured control.

Whereas high aggrecanase activity against the IGD cleavage site, as seen by Western blotting, was observed in cartilage stimulated by mechanical injury + TNF-α, no MMP activity against the IGD was observed in this condition during the 6 days of culture. This novel finding suggests that the activated proteolytic pathways are different between the TNF-α-stimulated cartilage and when cartilage is cultured alone, mechanically traumatized, or coincubated with SJC.

Activation of inflammatory pathways may be involved in the pathogenesis of OA and has been shown to associate with pain and to be prognostic of OA progression [14, 39]. As this study and others have shown, the synovial cells and/or other cells of the fibrous joint capsule may induce aggrecan cleavage by producing proteases themselves and could, by producing inflammatory mediators, induce chondrocytes to produce aggrecanases and MMPs [23, 26]. In the sequelae of severe joint injury, the activation of Toll-like receptors (TLRs) by matrix fragments may induce a vicious cycle by downstream activation of inflammation pathways [40, 41]. Aggrecan fragments may also contribute to continued activation of inflammatory pathways, and a 32-mer aggrecan peptide was recently shown to have antianabolic, procatabolic, and proinflammatory bioactivity mediated through TLR2 and nuclear factor-κB [42]. Importantly, with regard to the findings of the present study, cells other than synoviocytes (leukocytes, macrophages, fibroblasts, and endothelial cells) reside in the matrix underlying the synovial lining and could contribute to increased protease activity in the cartilage explant in vitro system [43, 44].

Our results extend previous findings suggesting that cross talk between cartilage and SJC could increase aggrecanase activity in the cartilage [23, 45] by showing that coincubation of mechanically injured cartilage with SJC increases both MMP and aggrecanase activity. We also found that the SJC produces active aggrecanases and MMPs. Our study further extends previous investigations by showing that the coincubation of cartilage with SJC induces not only aggrecan cleavage in the IGD (generating ARGS-CS2 fragments) [45, 46] but also proteolytic cleavage between CS2 and G3 (generating ARLE-G3 fragments), and leads to a higher degree of cleavage in the CS2 region (generating G1-KEEE fragments). This is important, considering that aggrecan degradation in this explant study, and in other in vitro studies [38, 47], starts with cleavage in the CS region followed by cleavage in the IGD.

Interestingly, the findings of the present study indicate that mechanical injury to cartilage alone, previously shown to induce increased MMP and aggrecanase gene expression (250-fold and 40-fold, respectively) [25], did not increase aggrecanase-mediated digestion of aggrecan. This novel observation suggests that additional soluble factors from the SJC are essential to increasing aggrecanase degradative activity toward aggrecan in the cartilage. We also found evidence of more MMP activity in mechanically injured cartilage with or without coincubation with SJC compared with cartilage cultured alone. These findings are consistent with previous studies which have shown that the combination of mechanical injury of cartilage plus incubation with inflammatory cytokines led to the release of significantly greater amounts of sGAG and specific mass spectrometry-detected matrix fragments (e.g., aggrecan, collagen III, cartilage oligomeric matrix protein) from the cartilage than cytokine treatment alone [27, 48]. Taken together, the findings of the present study are consistent with the hypothesis that mechanical injury alone does not induce or alter the mechanisms underlying aggrecan proteolysis but can affect the rate of release of the resulting fragments from the tissue via altered transport/diffusion. Also, the increased MMP activity observed in the mechanically injured cartilage coincubated with SJC could be related to increased access to the cartilage for proinflammatory molecules and proteases released from the SJC [27].

In contrast, when mechanically injured cartilage was cultured in the presence of TNF-α, only aggrecanase and no MMP activity was detected. These findings are interesting and could imply that different intracellular pathways for inducing aggrecan degradation are activated, depending on if cartilage is stimulated by SJC or by TNF-α. Such differences may be crucial when interpreting results from in vitro studies and when assessing the physiological relevance of any given model.

These findings are furthermore consistent with previous in vitro and in vivo investigations showing that high concentrations of catabolic cytokines such as TNF-α lead to early (8 h to 2 days) aggrecanase activity [49, 50]. In agreement with Madsen et al. [50], who used a similar bovine explant system, we did not detect MMP-generated FFGV fragments in the medium or in the cartilage during the 6 days of cartilage explant culture in the presence of TNF-α. In all other conditions, FFGV-aggrecan was detected as early as 1–2 days after incubation started. More complex systems, such as in vivo lipopolysaccharide-induced joint inflammation or acute knee injury, have implicated increased MMP activity toward type II collagen within 1 day of insult [15, 20, 51, 52]. However, whether MMP activity toward aggrecan is increased in these conditions is not well understood [53]. Studies designed to identify which factors released from the SJC increase aggrecanase and MMP activity are warranted. If these factors could be singled out, then they could represent novel targets for treatment of PTOA or joint injury.

There are several limitations to the present investigation. Owing to the small study sample size (three animals), our findings need to be repeated. Of note, the excision of SJC before culture traumatizes the SJC tissue, and resident cells may respond to this injury with an inflammatory phenotype. This is also applicable to the cartilage. We must also keep in mind that very young bovine cartilage was used in the experiment, and we do not know if the pattern of proteolysis is similar in older bovine cartilage or in adult human cartilage, even though the aggrecan fragment pattern observed in the bovine medium resembles the pattern seen in synovial fluid from patients with OA (Additional file 1: Figure S1). However, using this young bovine system of very tightly controlled age, we have previously shown that animal-to-animal variations in cartilage behavior are typically no larger than specimen-to-specimen variations within a single animal, giving us confidence that the results presented are meaningful. A strength of the present study is that we were able to follow a course of events using different experimental conditions, using well-characterized bovine aggrecan fragments rather than observing momentary time frames of the joint milieu, as is the case in analysis of joint fluid after, for example, a joint injury.

## Conclusions

The time-dependent release of bovine aggrecan fragments from cartilage explants during different culture conditions revealed striking differences related to activation of different proteolytic pathways by the presence of SJC or TNF-α. On the whole, our findings are consistent with the important involvement of the SJC in joint cartilage aggrecan cleavage. The SJC may induce aggrecan cleavage in the mechanically injured cartilage by producing both aggrecanases and MMPs, whereas exogenous TNF-α leads to aggrecan cleavage by activating aggrecanase and inhibiting MMP activity. The cells of the synovium and fibrous joint capsule may also secrete active molecules (e.g., cytokines) that can stimulate cartilage-originated aggrecanase and MMP activity, implicating that the cartilage coculture system including SJC may reveal additional physiologically relevant matrix degradation mechanisms associated with joint injury.

## Additional files

Additional file 1: Descriptive information on bovine aggrecan fragments detected by Western blot analysis. **Figure S1** Bovine aggrecan fragments detected by Western blot analysis. (PDF 353 kb)

Additional file 2: Figure S2. Anti-FFGV (**a**), anti-ARGS (**b**), anti-KEEE (**c**), and anti-G3 (**d**) Western blotting of medium samples. (PDF 647 kb)

Additional file 3: Table S1. The amount of aggrecan fragments released into medium. (PDF 79 kb)

## Abbreviations

ADAMTS: A disintegrin and metalloproteinase with thrombospondin motifs
CS: Chondroitin sulfate region
DMMB: Dimethylmethylene blue
EDTA: Ethylenediaminetetraacetic acid
KS: Keratan sulfate region
IGD: Interglobular domain
IgG: Immunoglobulin G
MMP: Matrix metalloproteinase
OA: Osteoarthritis
PTOA: Posttraumatic osteoarthritis
SB: Sample buffer
sGAG: Sulfated glycosaminoglycan
SJC: Synovium + joint capsule
TLR: Toll-like receptor
TNF-α: Tumor necrosis factor-α
