# Supplementary material for: Coculture of bovine cartilage with synovium and fibrous joint capsule increases aggrecanase and matrix metalloproteinase activity
Source: Arthritis Res Ther. 2017 Jul 5;19:157. doi: 10.1186/s13075-017-1318-9 (PMC5498889; doi:10.1186/s13075-017-1318-9)
Supplement: Supplementary file 1 — Descriptive information on bovine aggrecan fragments detected by Western blot analysis. Figure S1 Bovine aggrecan fragments detected by Western blot analysis. (PDF 353 kb) [file 13075_2017_1318_MOESM1_ESM.pdf]

Additional file 1

Bovine cartilage co-cultured with synovium and fibrous joint capsule increases aggrecanase and matrix metalloproteinase activity

Per Swärd, Yang Wang, Maria Hansson, L Stefan Lohmander, Alan J. Grodzinsky, André Struglics

Bovine aggrecan fragments detected by Western blot

Aggrecan fragments were detected using Western blot in the medium and cartilage explants and after ADAMTS-4 and MMP-3 digestion of purified bovine aggrecan *in vitro* (Fig. A1). Here we present all the detected bovine aggrecan fragments regardless of treatment condition.

MMP cleaved aggrecan generating FFGV fragments were detected in the medium

In the cartilage medium three FFGV fragments were detected: 389, 300, and 90 kDa (Fig. A1a). Aggrecan purified from bovine cartilage (A1D1 fraction) also contained a weak 389 kDa FFGV fragment, while the cartilage plugs after 16 days of culture did not contain any FFGV fragments (Gu-PG, Fig. A1a). No FFGV fragments were detected in the synovium-joint capsule or in the medium from the synovium-joint capsule (data not shown). The C-terminal end of the 389, 300 and 90 kDa FFGV fragment were estimated using a calculation model [Struglics et al. Osteoarthritis Cartilage 2006;14:898-905] to be localized within the G3 domain, the chondroitin sulfate (CS) region 2 and the keratan sulfate (KS) region, respectively.

Aggrecanase cleaved aggrecan generating ARGS fragments were detected in the medium

In the bovine cartilage medium a 296 kDa ARGS fragment was detected (Fig. A1b). Aggrecan purified from bovine cartilage (A1D1 fraction) did not contain any ARGS fragments, and except for condition E (mechanical injury + TNFα), neither did the cartilage plugs after culturing (Fig. A1b). No ARGS fragments were detected in the synovium-joint capsule or in the medium from the synovium-joint capsule (data not shown). The C-terminal end of the 296 kDa ARGS fragment was estimated (using a calculation model [Struglics et al. Osteoarthritis Cartilage 2006;14:898-905]) to be within the CS2 region. Due to differences in molecular weight seen by Western blot, it was confirmed that this fragment does not have the sequence -KEEE as C-terminal (Fig. A1c), and it is more likely that the 296 kDa bovine ARGS fragment is ARGS-GELE where the C-terminal was generated by aggrecanase cleavage in the GELE/GRGT site (Fig. A1b). A corresponding ARGS fragment has been verified by Western blot as ARGS-SELE (310 kDa) in human OA synovial fluid and by *in vitro* ADAMTS-4 digestion of purified human aggrecan [Struglics et al. Osteoarthritis Cartilage 2006;14:101-13, Struglics et al. Osteoarthritis Cartilage 2011;19:1047-57].

Aggrecanase cleaved aggrecan generating KEEE fragments were detected in medium and cartilage

In bovine cartilage medium seven KEEE fragment were detected; 329, 240, 175, 159, 136, 79 and 64 kDa; while in cartilage plugs only the 329, 240, 136 and the 64 kDa KEEE fragments were found (Fig. A1d). Similar KEEE fragments were also found in the synovium-joint capsule tissue and medium (condition F, data not shown). The 329 and 64 kDa KEEE fragments were identified, with G1 and GRGT antibodies, as G1-KEEE and GRGT-KEEE respectively (data not shown), and by *in vitro* ADAMTS-4 digestion of purified bovine aggrecan (Fig. A1d). The N-terminal ends of the 240, 175, 159, 136 and 79 kDa KEEE fragments were estimated (using a calculation model [Struglics et al. Osteoarthritis Cartilage 2006;14:898-905]) to be within the KS and CS regions, where the N-terminal cuts in the CS1 region for the 136, 159 and 175 kDa KEEE fragments are suggested to be generated by MMPs.

Aggrecan G3 fragments were detected in medium and cartilage

In bovine cartilage medium seven G3-fragments were detected: > 400, 228, 182, 139, 105, 38 and 30 kDa; while in the cartilage plugs of these fragments only the >400 kDa G3 fragment was found (Fig. A1e). None of these G3 fragments were detected in the joint synovium-capsule tissue or in the medium from the synovium-joint capsule (data not shown). The >400 kDa G3 fragment was identified with a G1 antibody as full-length aggrecan, and the N-terminal of the 139, 105 and 38 kDa G3 fragments were identified as AGEG, LGQR and ARLE by Western blot and neoepitope antibodies, respectively (data not shown), verifying that these are AGEG-G3 , LGQR-G3 and ARLE-G3 fragments (Fig. A1e). The N-terminals of the 228 and 182 kDa G3 fragments was not identified by neoepitope antibodies, although ADAMTS-4 *in vitro* proteolysis of bovine aggrecan renders fragments of similar molecular weight as found in the explants medium, and thus strengthen the assumption that these fragments are GRGT-G3 and GLGS-G3, respectively (Fig. A1e).

Bovine and human aggrecan fragments

Based on their molecular weight, almost all of the bovine aggrecan fragments found in the medium after incubation in the explant cultures have corresponding fragments found in the synovial fluid of OA patients (Fig. A1). However, bovine medium revealed a 38 kDa ARLE-G3 fragment (Fig. A1d) while the corresponding human fragment THLE-G3 has not been found *in vivo* [Struglics & Hansson Biochem J 2012;446:213-23]. Human synovial fluid contains a 120-160 kDa ARGS-CS1 fragment [Struglics et al. Osteoarthritis Cartilage 2011;19:1047-57] that was not detected in the bovine medium (Fig. A1b).

Fig. S1

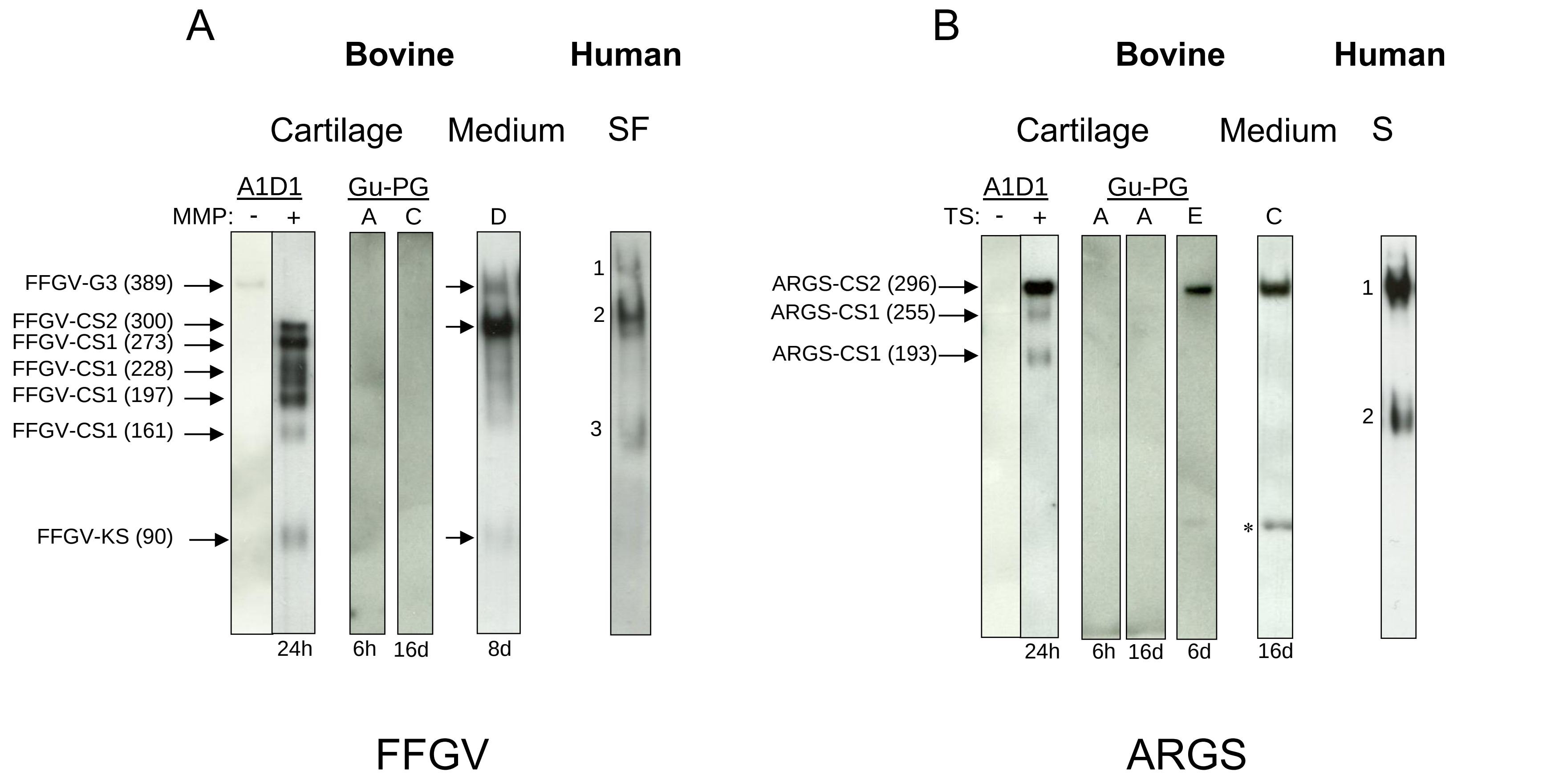

A
Bovine
Human
Cartilage
Medium
SF
A1D1
Gu-PG
MMP: - +
A C
D
FFGV-G3 (389)
FFGV-CS2 (300)
FFGV-CS1 (273)
FFGV-CS1 (228)
FFGV-CS1 (197)
FFGV-CS1 (161)
FFGV-KS (90)
1
2
3
24h
6h
16d
8d
FFGV
B
Bovine
Human
Cartilage
Medium
S
A1D1
Gu-PG
TS: - +
A A E
C
ARGS-CS2 (296)
ARGS-CS1 (255)
ARGS-CS1 (193)
1
2
*
24h
6h
16d
6d
16d
ARGS

Fig. S1

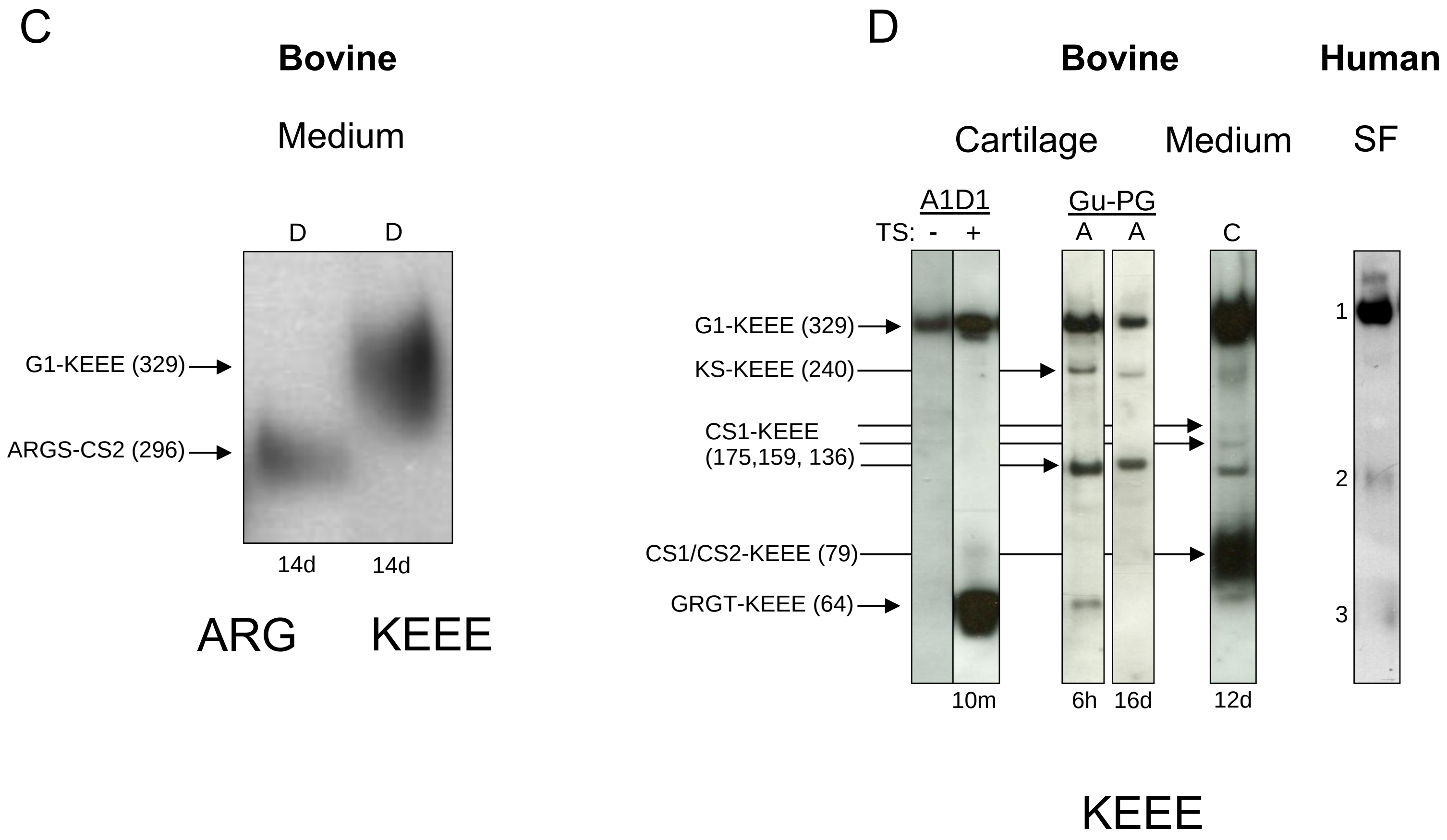
C
Bovine
Medium
D
D
G1-KEEE (329)
ARGS-CS2 (296)
14d
14d
ARG
KEEE
D
Bovine
Human
Cartilage
Medium
SF
A1D1
Gu-PG
TS: - +
A A
C
G1-KEEE (329)
KS-KEEE (240)
CS1-KEEE
(175,159, 136)
CS1/CS2-KEEE (79)
GRGT-KEEE (64)
1
2
3
10m
6h
16d
12d
KEEE

Fig. S1

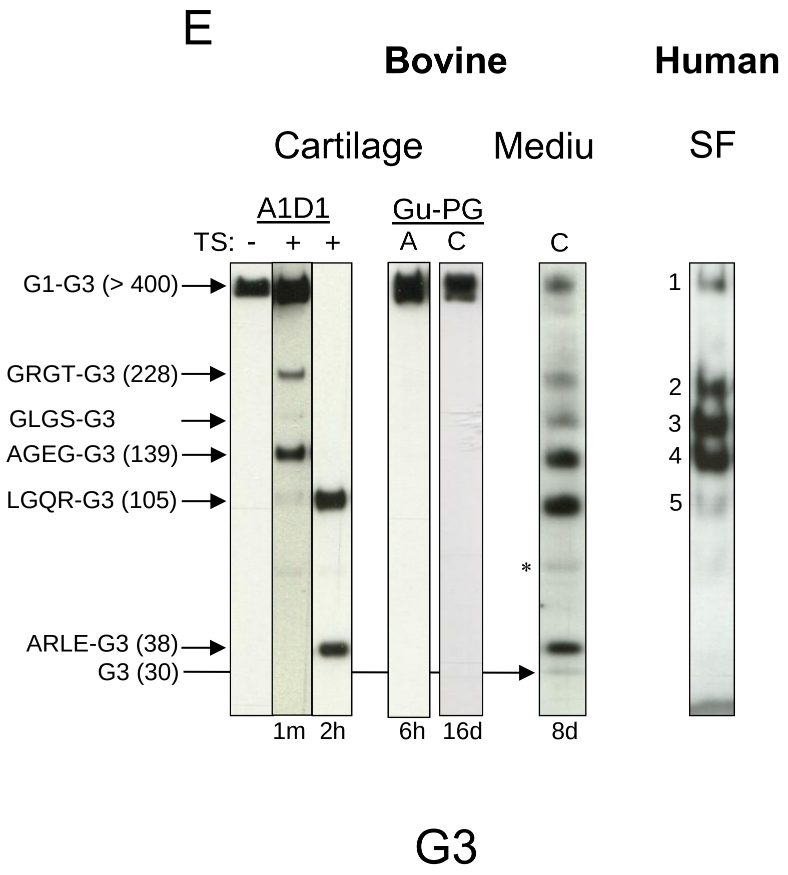
E
Bovine
Human
Cartilage
Mediu
SF
A1D1
Gu-PG
TS: - + +
A C
C
G1-G3 (> 400)
GRGT-G3 (228)
GLGS-G3
AGEG-G3 (139)
LGQR-G3 (105)
ARLE-G3 (38)
G3 (30)
*
1
2
3
4
5
1m 2h
6h 16d
8d
G3

Figure S1. Bovine aggrecan fragment pattern. Bovine immunobands (detected by Western blot using FFGV, ARGS, KEEE and G3 aggrecan antibodies) in aggrecan A1D1-fraction (purified from cartilage) and in samples from cartilage explant culture system (according to Material and Methods) are shown. The bovine explant system samples were harvested after 6h to 16 days of incubation in conditions A-E (Table 1), and guanidinium-extracted proteoglycans (Gu-PG) were purified from the cartilage plugs; Gu-PG and culture-medium were applied for Western blot. As a comparison, Western blot of human synovial fluid (SF) samples (D1 fraction prepared from a synovial fluid OA-pool [Struglics & Hansson Biochem J 2012;446:213-23]) are shown. All samples were deglycosylated and samples were run on 3-8% Tris-acetate SDS gels. Bovine aggrecan fragments discussed in the text are shown on the left side in each panel with the molecular mass in kDa indicated. +, bovine aggrecan A1D1-fraction incubated 1 min (1m), 10 min (10m), 2h and 24h with ADAMTS-4 (TS) or 24h with MMP-3 [Struglics et al. Osteoarthritis Cartilage 2006;14:101-13, Struglics & Hansson Biochem J 2012;446:213-23]. Except for panel-c, representative Western blots images from full-sized blotted gels are shown. Sample loading (per lane): A1D1 0.12-4 µg sGAG, A1D1 + ADAMTS-4 0.06-4 µg sGAG, A1D1 + MMP-3 6 ng sGAG, Gu-PG 2-2.4 µg sGAG, medium 40 µl, SF 1-3 µg sGAG. Molecular mass in kDa of human synovial fluid aggrecan fragments [Struglics et al. Osteoarthritis Cartilage 2011;19:1047-57, Struglics & Hansson Biochem J 2012;446:213-23]: FFGV band-1 (400), -2 (320, 330), -3 (130-190); ARGS band-1 (280-310), -2 (120-160); KEEE band-1 (340-370), -2 (124), -3 (50); G3 band-1 (>400), -2 (214), -3 (171), -4 (137), -5 (103). Bands marked by * are considered to be false-positive immunobands since they could not be blocked by their corresponding immunopeptides.
