# Supplementary material for: Coculture of bovine cartilage with synovium and fibrous joint capsule increases aggrecanase and matrix metalloproteinase activity
Source: Arthritis Res Ther. 2017 Jul 5;19:157. doi: 10.1186/s13075-017-1318-9 (PMC5498889; doi:10.1186/s13075-017-1318-9)
Supplement: Supplementary file 2 — Anti-FFGV (a), anti-ARGS (b), anti-KEEE (c), and anti-G3 (d) Western blotting of medium samples. (PDF 647 kb) [file 13075_2017_1318_MOESM2_ESM.pdf]

Additional file 2

Bovine cartilage co-cultured with synovium and fibrous joint capsule increases aggrecanase and matrix metalloproteinase activity

Per Swärd, Yang Wang, Maria Hansson, L Stefan Lohmander, Alan J. Grodzinsky, André Struglics

Fig. S2

(A) FFGV

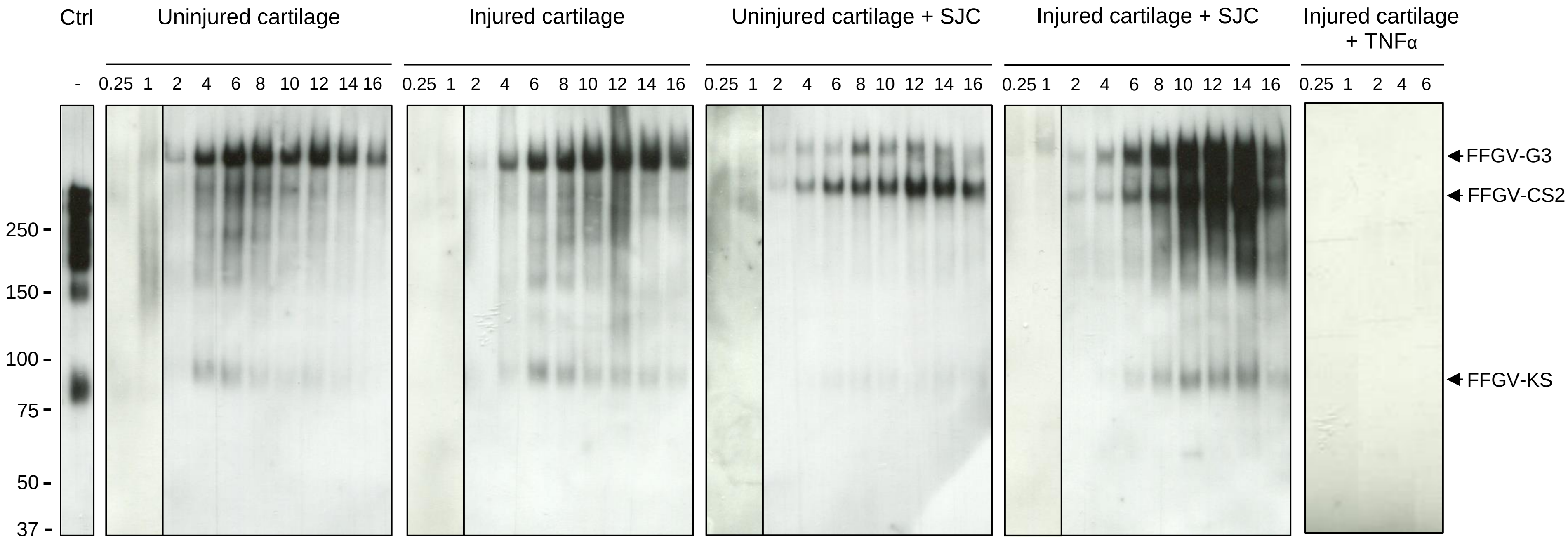

Fig. S2

(B) ARGS

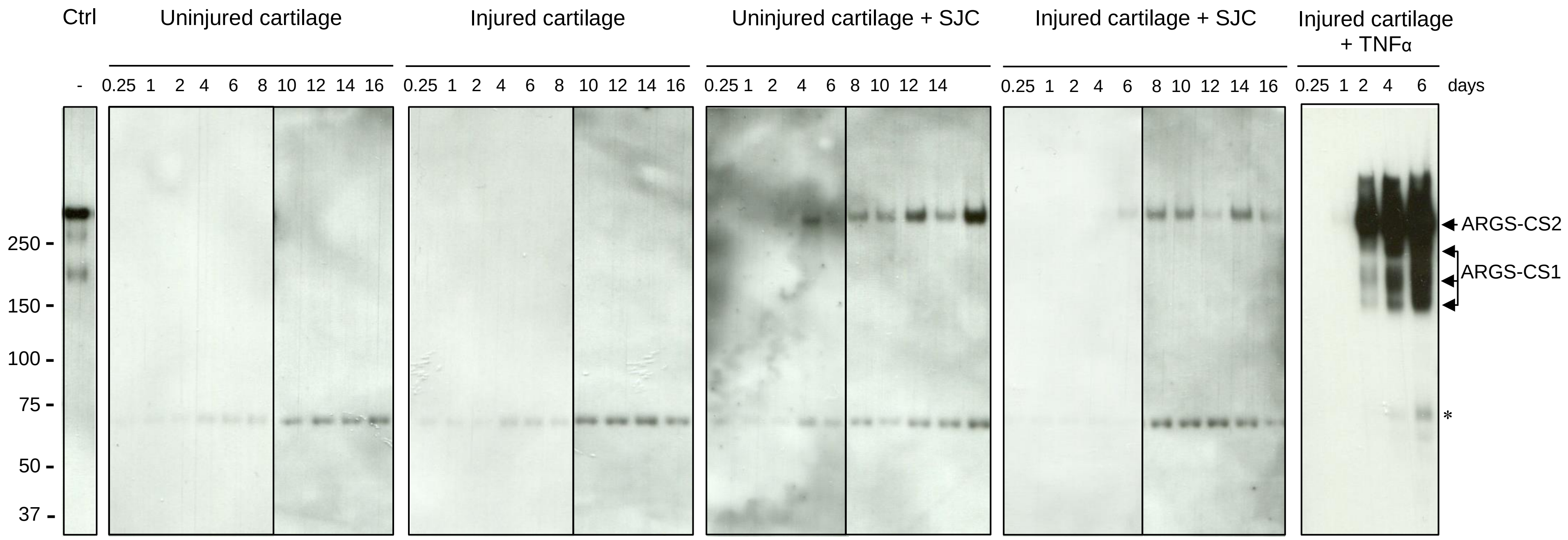

Ctrl
Uninjured cartilage
Injured cartilage
Uninjured cartilage + SJC
Injured cartilage + SJC
Injured cartilage + TNFα
- 0.25 1 2 4 6 8 10 12 14 16 0.25 1 2 4 6 8 10 12 14 16 0.25 1 2 4 6 8 10 12 14 0.25 1 2 4 6 8 10 12 14 16 0.25 1 2 4 6 days
250
150
100
75
50
37
ARGS-CS2
ARGS-CS1
*

Fig. S2

(C)

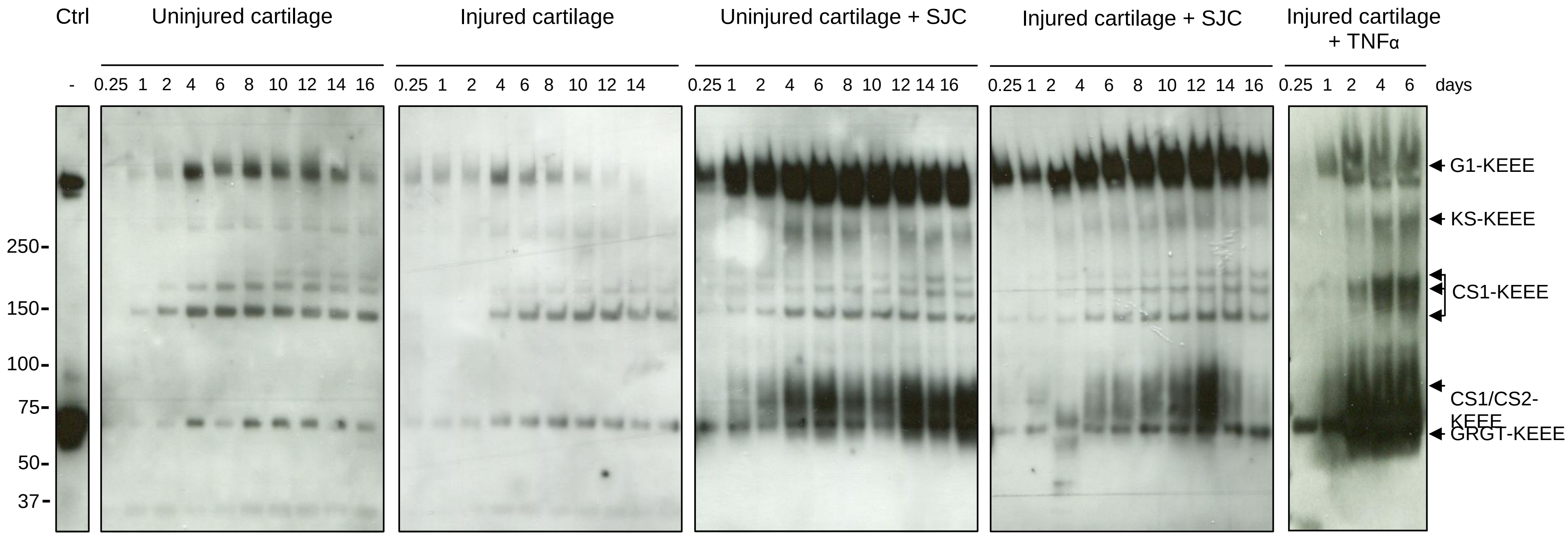

Ctrl
Uninjured cartilage
Injured cartilage
Uninjured cartilage + SJC
Injured cartilage + SJC
Injured cartilage + TNFα
- 0.25 1 2 4 6 8 10 12 14 16 0.25 1 2 4 6 8 10 12 14 0.25 1 2 4 6 8 10 12 14 16 0.25 1 2 4 6 8 10 12 14 16 0.25 1 2 4 6 days
250
150
100
75
50
37
G1-KEEE
KS-KEEE
CS1-KEEE
CS1/CS2-KEEE
GRGT-KEEE

Fig. S2

(D) G3

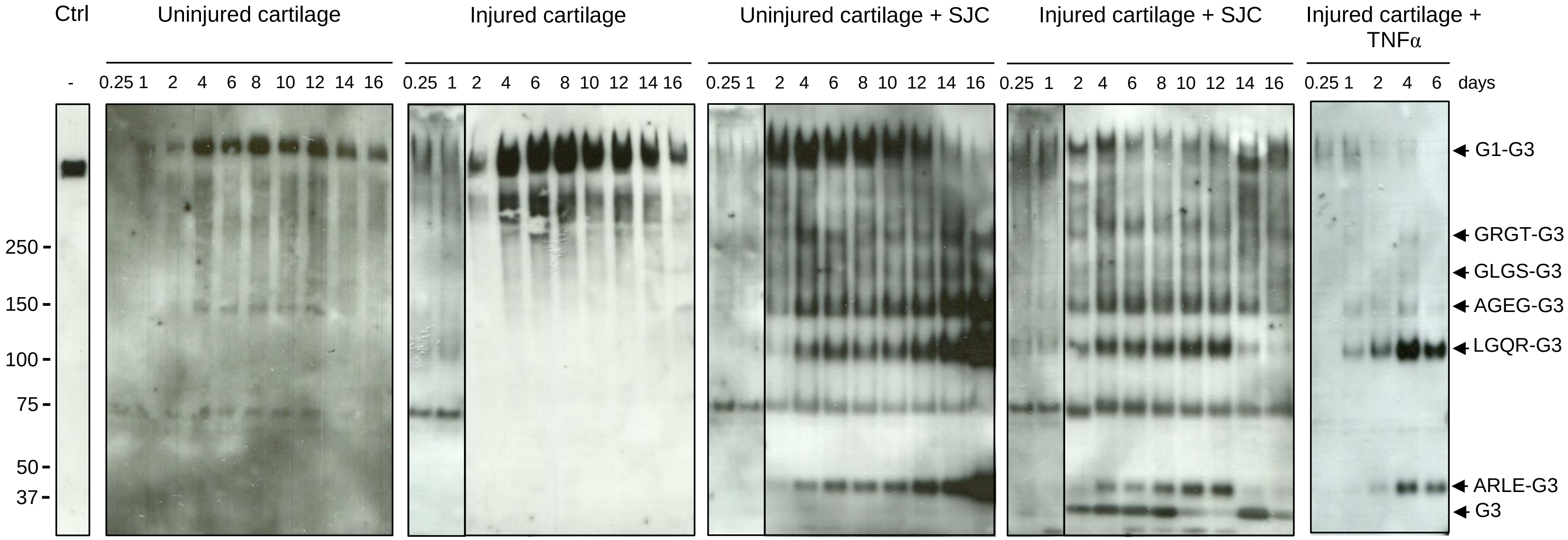

Figure A2. Anti-FFGV (A), -ARGS (B), -KEEE (C) and –G3 (D) Western blot of medium samples. Medium from cartilage explant cultures were collected at different time points (0.25 to 16 days), deglycosylated and run (40 – 53 µl medium/lane) on 3-8% Tris-acetate SDS-gels and applied for Western blot. Representative Western blots images from full-sized blotted gels are shown. Band marked by * is considered to be a false-positive immunoband since it could not be blocked by the corresponding immunopeptide. Ctrl, positive controls and standards: bovine aggrecan A1D1-fraction digested with MMP-3 for 24h (A), or with ADAMTS-4 for 24h (B), or with ADAMTS-4 for 10 min (C); bovine aggrecan A1D1-fraction (D). SJC, synovium-joint capsule.
