# Supplementary material for: Coculture of bovine cartilage with synovium and fibrous joint capsule increases aggrecanase and matrix metalloproteinase activity
Source: Arthritis Res Ther. 2017 Jul 5;19:157. doi: 10.1186/s13075-017-1318-9 (PMC5498889; doi:10.1186/s13075-017-1318-9)
Supplement: Supplementary file 3 — The amount of aggrecan fragments released into medium. (PDF 79 kb) [file 13075_2017_1318_MOESM3_ESM.pdf]

Additional file 3

Bovine cartilage co-cultured with synovium and fibrous joint capsule increases aggrecanase and matrix metalloproteinase activity

Per Swärd, Yang Wang, Maria Hansson, L Stefan Lohmander, Alan J. Grodzinsky, André Struglics

Table S1. The release of aggrecan fragments into medium. Cartilage explants from the patellofemoral groove of six knees from three cows were cultured during 16 days. The mean accumulated amounts of total FFGV and ARGS-CS2 fragments, and G1-KEEE and G1-G3 aggrecan fragments from the two knees per cow is presented for each cow at each time-point for the analyzed conditions: A = uninjured cartilage, B = mechanically injured cartilage, C = uninjured cartilage co-cultured with synovium-joint capsule, D = mechanically injured cartilage co-incubated with synovium-joint capsule. AU, arbitrary units.

**FFGV** (ng/mg sGAG)		**Condition A**			**Condition B**			**Condition C**			**Condition D**		
		**Cow**			**Cow**			**Cow**			**Cow**		
	**Days**	**1**	**2**	**3**	**1**	**2**	**3**	**1**	**2**	**3**	**1**	**2**	**3**
	0.25	31.81	28.91	17.92	34.25	20.45	17.88	39.15	23.37	28.94	64.87	65.04	83.99
	1	62.24	47.77	35.85	67.01	40.90	35.76	64.68	59.21	47.82	129.74	114.91	167.99
	2	62.24	56.57	53.77	78.93	72.26	63.18	90.21	70.11	75.51	179.47	147.43	232.38
	4	82.98	104.33	99.17	101.27	102.26	89.41	127.66	82.58	123.33	244.34	195.12	293.98
	6	103.73	169.70	152.94	123.60	154.07	196.70	345.52	93.49	179.96	374.08	244.99	358.37
	8	207.45	226.27	188.79	178.71	204.51	224.12	714.88	116.86	236.59	536.25	310.03	419.97
	10	269.69	255.18	224.63	257.63	265.86	277.76	1162.52	129.32	274.34	893.03	440.11	526.36
	12	311.18	282.83	260.48	357.41	327.22	313.53	1596.55	176.06	312.09	1476.85	780.49	629.95
	14	331.93	301.69	296.32	424.43	379.03	447.04	2005.06	199.44	349.85	1606.59	1025.47	1301.90
	16	342.99	311.74	322.61	481.02	409.02	482.81	2399.94	222.81	387.60	1898.50	1203.25	1450.29

ARGS-CS2 (ng/mg sGAG)	Condition A			Condition B			Condition C			Condition D		
	Cow			Cow			Cow			Cow		
Days	1	2	3	1	2	3	1	2	3	1	2	3
0.25	0.00	0.00	0.00	0.00	0.00	0.00	0.00	0.00	0.00	0.00	0.00	0.00
1	0.00	0.00	0.00	0.00	0.00	0.00	0.00	0.00	0.00	0.00	0.00	0.00
2	0.00	0.00	0.00	0.00	0.00	0.00	0.00	0.00	0.00	0.00	0.00	0.00
4	0.00	0.00	0.00	0.00	0.00	0.00	0.00	0.00	0.00	0.00	0.00	0.00
6	0.00	0.00	0.00	0.00	0.00	0.00	0.00	0.00	0.00	0.00	0.00	1030.32
8	0.00	0.00	0.00	0.00	0.00	0.00	0.00	316.29	0.00	730.86	292.68	1786.26
10	0.00	0.00	0.00	0.00	0.00	0.00	0.00	550.01	0.00	1070.34	570.19	1786.26
12	0.00	0.00	0.00	0.00	0.00	0.00	0.00	830.46	330.97	1070.34	570.19	1786.26
14	0.00	0.00	0.00	0.00	0.00	0.00	0.00	830.46	1350.31	1070.34	845.53	2729.79
16	0.00	0.00	0.00	0.00	0.00	0.00	0.00	1378.91	3426.73	1070.34	845.53	2729.79

G1-KEEE (AU/mg sGAG)	Condition A			Condition B			Condition C			Condition D		
	Cow			Cow			Cow			Cow		
Days	1	2	3	1	2	3	1	2	3	1	2	3
0.25	0.01	0.03	0.01	0.18	0.04	0.01	0.14	0.13	0.05	1.77	0.86	0.73
1	0.05	0.08	0.01	0.28	0.07	0.01	0.48	0.47	0.16	2.52	1.37	1.23
2	0.12	0.15	0.02	0.46	0.13	0.01	0.54	1.03	0.50	4.01	2.35	2.07
4	0.26	0.30	0.08	0.90	0.26	0.03	2.24	2.28	2.32	11.50	5.12	4.00
6	0.36	0.49	0.12	1.15	0.37	0.04	3.51	3.80	3.47	20.08	7.89	6.25
8	0.45	0.86	0.17	1.34	0.47	0.06	4.52	5.18	4.37	24.00	10.14	8.64
10	0.54	1.10	0.21	1.58	0.54	0.08	5.02	6.32	5.05	28.14	12.38	10.71
12	0.73	1.31	0.26	1.84	0.62	0.10	6.44	7.39	5.76	32.78	15.51	13.02
14	0.83	1.41	0.29	2.01	0.70	0.10	7.96	8.16	6.97	37.05	16.81	14.93
16	0.91	1.51	0.31	2.12	0.74	0.10	8.87	8.83	10.20	41.04	18.38	15.94

G1-G3 (ng/mg sGAG)	Condition A			Condition B			Condition C			Condition D		
	Cow			Cow			Cow			Cow		
Days	1	2	3	1	2	3	1	2	3	1	2	3
0.25	138.3	0.0	358.5	446.8	136.3	1192.1	0.0	0.0	377.5	216.2	0.0	2799.8
1	276.6	0.0	1194.8	1489.2	136.3	1192.1	0.0	0.0	1258.4	432.5	0.0	2799.8
2	0.0	1257.0	1194.8	1489.2	1363.4	2384.2	170.2	1558.1	2516.9	2162.3	2168.0	2799.8
4	1383.0	3771.1	3584.5	2978.4	8180.5	4768.5	1702.1	1558.1	3775.3	2162.3	6504.1	5599.6
6	4149.1	6285.2	5974.2	2978.4	14997.5	8344.8	3404.2	3116.2	5033.8	4324.6	8672.1	5599.6
8	5532.1	10056.3	8363.9	4467.6	21814.6	11921.2	5106.3	4674.3	6292.2	4324.6	13008.1	8399.4
10	8298.2	12570.3	9558.7	4467.6	28631.6	14305.4	6808.3	6232.4	8809.1	4324.6	15176.2	8399.4
12	11064.2	15084.4	11948.4	4467.6	35448.7	16689.6	8510.4	7790.5	10067.5	6486.9	21680.2	8399.4
14	12447.3	17598.4	14338.1	5956.9	40902.3	19073.8	10212.5	7790.5	10067.5	8649.2	28184.3	11199.1
16	12447.3	18855.5	15533.0	7446.1	43629.1	20266.0	10212.5	7790.5	11326.0	8649.2	34688.3	11199.1
